# Electrical Detection of Spin-Hall-Induced Auto-oscillations in Lithium Aluminate Ferrite Thin Films

**DOI:** 10.1021/acs.nanolett.4c06305

**Published:** 2025-04-10

**Authors:** Haowen Ren, Ya-An Lai, Sanyum Channa, Daisy A. O’Mahoney, Xin Yu Zheng, Yuri Suzuki, Andrew D. Kent

**Affiliations:** † Center for Quantum Phenomena, Department of Physics, New York University, New York, New York 10003, United States; ‡ Department of Physics, Stanford University, Stanford, California 94305, United States; § Geballe Laboratory for Advanced Materials, Stanford University, Stanford, California 94305, United States; ∥ Department of Materials Science and Engineering, Stanford University, Stanford, California 94305, United States; ⊥ Department of Applied Physics, Stanford University, Stanford, California 94305, United States

**Keywords:** magnonics, spin-Hall nanooscillators, spin oscillators, ferrimagnetic insulators, spin-Hall effect, ferromagnetic resonance

## Abstract

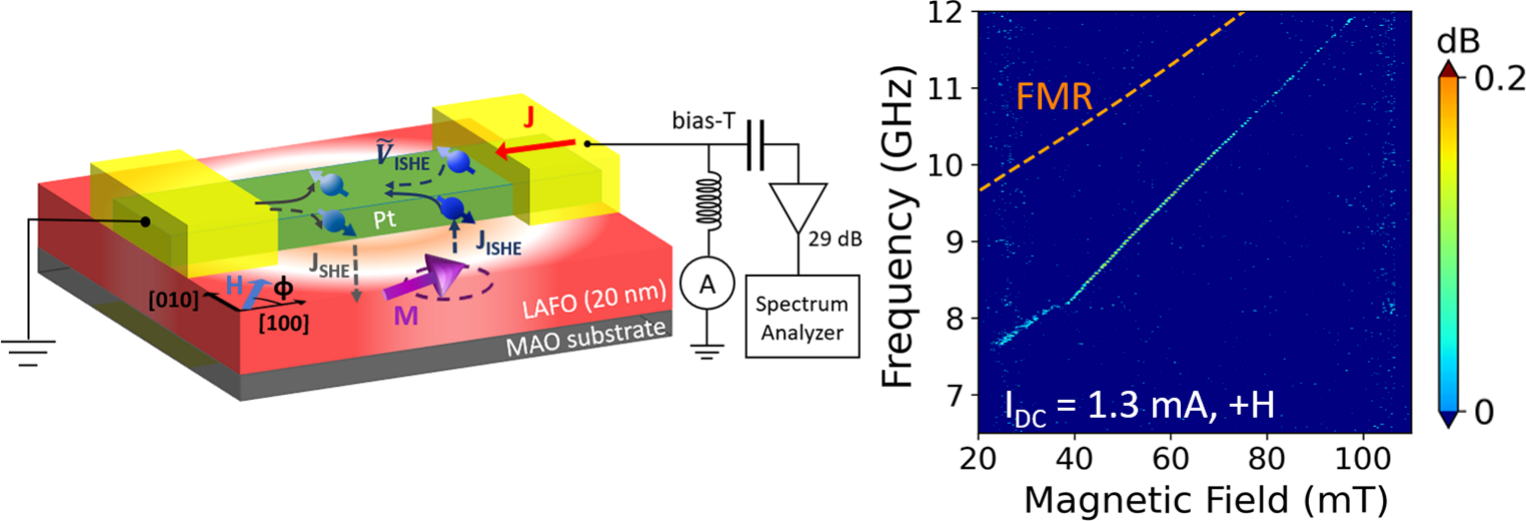

Ferrimagnetic insulators with ultralow damping are of great interest for their potential applications in energy-efficient computing devices. Here, we report the direct electrical detection of magnetic auto-oscillations in unpatterned ultralow damping ferrimagnetic insulator epitaxial Li_0.5_Al_0.5_Fe_2_O_4_ thin films, driven by a current in a proximal Pt nanowire. Auto-oscillations occur for only one current polarity, consistent with the spin-Hall effect inducing the oscillation state. Micromagnetic modeling shows good agreement with the experimental frequency and field dispersions, showing only one dominant oscillation mode, in contrast to the multiple modes typically observed in transition-metal nanowire-type spin-Hall nanooscillators. This study illustrates a new material system for neuromorphic computing and magnonics, a simple material platform with the direct-current generation of high-frequency (∼10 GHz) signals and their electrical detection.

Spin-Hall nanooscillators (SHNOs) have drawn tremendous attention due to their potential in neuromorphic computing,^1−3^ magnonics,^4,5^ and Ising machines.^6,7^ Many different types of nanooscillators have been achieved with different device geometries and materials, as well as physical mechanisms of excitation and detection.^8−10^ Within these different approaches, SHNOs stand out as one of the most promising devices due to their ease of fabrication, low current and power operation, and potential for scaled-down coupled devices for energy-efficient computing architectures.^11−17^

In SHNOs, auto-oscillation via electrical current application can induce spin-wave excitations. Magnetic materials with low damping are one of the keys to enabling the auto-oscillation state because they can reduce energy losses and increase spin torque efficiency. This is because current-induced spin torques compete against the damping torques to induce magnetization oscillations. Among different materials, ferrimagnetic insulators exhibit one of the lowest dampings due to a lack of conduction electrons. They are thus favorable materials for nanooscillator applications compared to conventional transition-metal magnetic materials.^18^

In magnetic thin films, there are multiple available nonlinear magnon scattering channels. Energy distributed in multiple channels can inhibit magnetic auto-oscillations because nonlinear coupling between spin-wave modes may limit the amplitude of any single mode to below the detectable threshold.^19,20^ One method of overcoming this is through geometrical confinement. By reducing the lateral dimension of a ferrimagnetic thin film^13^ or adding a magnetic element on top of it,^21^ one can create a potential well for spin waves with discrete spin-wave spectra. Spin-wave confinement reduces the scattering between spin-wave modes and can lead to more favorable conditions for auto-oscillation excitation.

Previous research has successfully demonstrated auto-oscillations in ferrimagnetic insulator yttrium iron garnet (YIG) in geometric-constrained designs, including nanodisk and nanowire geometries.^22−26^ The auto-oscillation signals were detected optically by Brillouin light scattering spectroscopy,^24,26^ electrically and inductively,^23^ the latter by a near-device antenna.

In this study, we demonstrate the spin-Hall-effect-induced auto-oscillations in a new epitaxial ferrimagnetic insulator, lithium aluminum ferrite (LAFO) thin films.^27,28^ We report the successful electrical detection of coherent auto-oscillation signals based on the ISHE. These findings provide a new way to fabricate high-efficiency SHNOs on extended ferrimagnetic insulator thin films, in contrast to the nanowire and nanodisk geometry YIG structures studied to date. Furthermore, LAFO has tunable magnetic properties, such as magnetic anisotropy and saturation magnetization, by varying the composition of Al and Fe and the strain state by the choice of substrate.^28,29^ This allows the design of new types of magnonic devices.^30^

Conventional SHNOs use transition-metal magnetic materials to enable the electrical detection of oscillation signals, typically using anisotropic magnetoresistance (AMR), giant magnetoresistance, and tunneling magnetoresistance as signal sources.^31^ In general, the amplitude of these signal sources is strong enough to be detected by a spectrum analyzer. In heavy-metal/ferrimagnetic insulator systems, electrical signals mainly originate from spin-Hall magnetoresistance (SMR) associated with the ISHE. However, direct electrical detection of such weak signals is difficult because the output power can be close to the Johnson noise floor. Thus, as noted earlier, the oscillation signals in such heavy-metal/ferrimagnetic insulator systems have been detected by other means.^23,24,26,32^

Figure 1a shows the SMR signal of a Pt(6 nm)/LAFO(20 nm) heterostructure, as a function of the angle ϕ between the applied charge current and the external field *H*_ext_ directions in the film plane. The angular dependence of SMR can be described by cos ϕ, which is consistent with expectations. The magnitude of the SMR signal, Δ*R* = 0.008%, is at least 2 orders lower than the AMR signal in ferromagnetic metals and is comparable to previously reported SMR values in heavy-metal/YIG heterostructures.^33,34^

**Figure 1:**
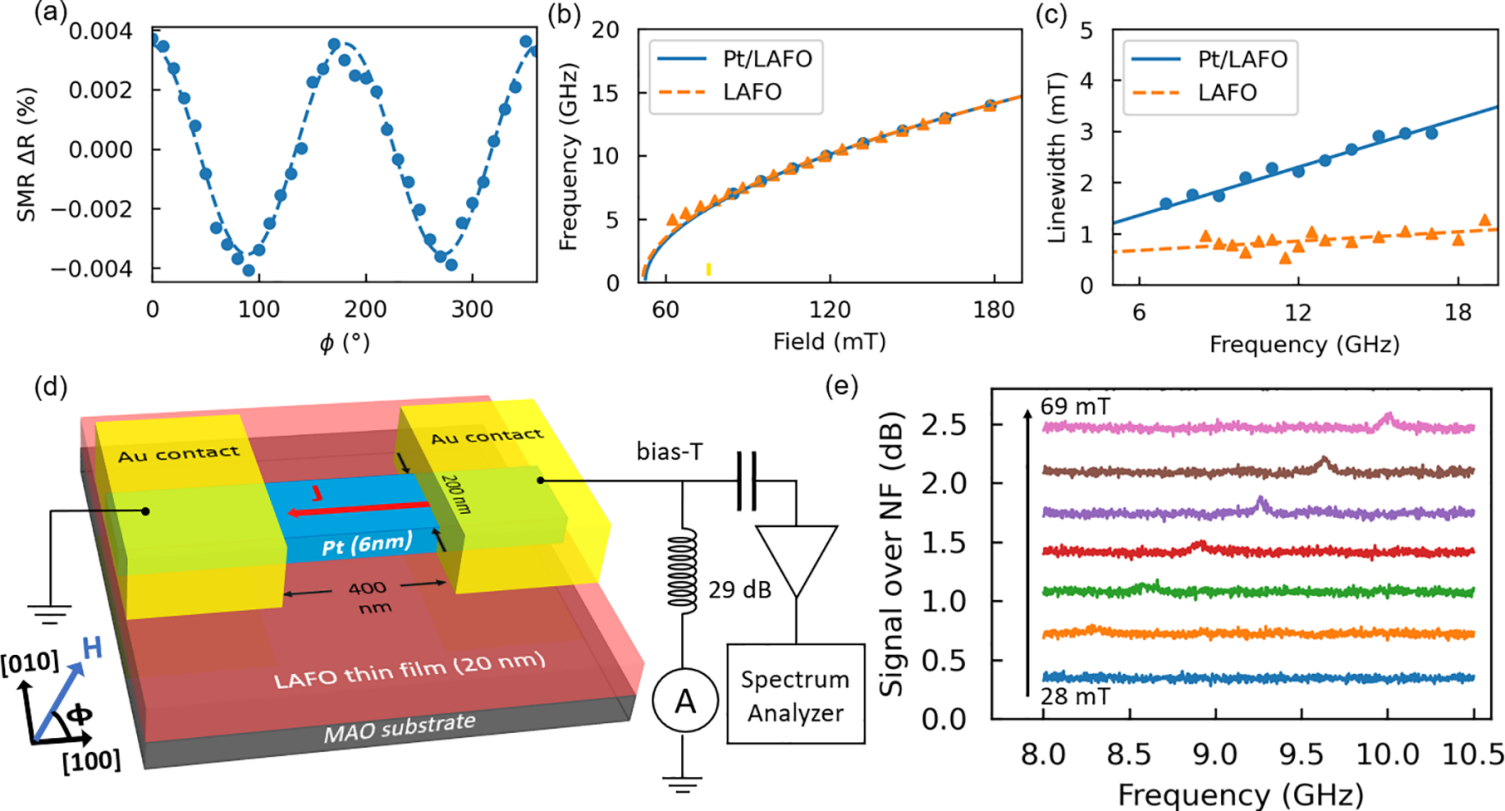
(a) Change in resistance associated with SMR as a function of the angle between the external magnetic field of 200 mT and current, conducted on a micron (2 × 5.8 μm^2^)-scale wire. (b) FMR measurements of Pt/LAFO and LAFO thin films with an in-plane *H*_ext_ applied along the [100] LAFO hard axis. (c) Frequency dependence of the FMR linewidth for applied fields that saturate the LAFO magnetization. (d) Schematic of the Pt/LAFO nanowire device and spectrum analyzer measurement setup. (e) Measured auto-oscillation signal as a function of the frequency at a fixed current of 1.4 mA, with *H*_ext_ varying from 28 to 69 mT at an angle ϕ = 70° to the current.

Ferromagnetic resonance spectroscopy (FMR) measurements are conducted on an as-deposited LAFO and Pt/LAFO thin film with *H*_ext_ applied along [100]. The dispersion relationships of Pt/LAFO and bare LAFO films are shown in Figure 1b. The data are fit to the Kittel model, , where μ_0_ is the vacuum permeability, γ is the gyromagnetic ratio, *H*_ext_ is the external magnetic field, and *H*_a_ is the in-plane anisotropy field. We note that LAFO thin films have a magnetocrystalline anisotropy with an easy axis along the ⟨110⟩ directions and a hard axis along the ⟨100⟩ directions.^16,28^ The results show that the FMR frequency versus applied field dispersion is not modified after Pt deposition. We find μ_0_*M*_eff_ = 1841.8 ± 2.7 mT for bare LAFO films and μ_0_*M*_eff_ = 1845.3 ± 4.6 mT for Pt/LAFO thin films. We also extract μ_0_*H*_a_ = −47.1 ± 0.6 mT for bare LAFO films and μ_0_*H*_a_ = −51.8 ± 2.0 mT for Pt/LAFO thin films. Figure 1c shows the FMR linewidth versus frequency. The zero-frequency intercept of a linear fit to the data gives an inhomogeneous broadening μ_0_Δ*H*_0_ of 0.48 ± 0.15 and 0.53 ± 0.11 mT before and after Pt deposition, respectively. The slope of the fit shows that the Gilbert damping constant increases from α_LAFO_ = (8.7 ± 0.3) × 10^–4^ to α_Pt/LAFO_ = (4.2 ± 0.3) × 10^–3^ after Pt deposition. This increase in damping is associated with the spin pumping effect; FMR excitation leads to the injection of spin current and loss of spin angular momentum in Pt. To quantify the spin injection across the Pt/LAFO interface, we compute the spin-mixing conductance using *G*_↑↓_ = *G*_0_Δα(*M*_s_*t*_LAFO_/*g*μ_B_), where *G*_0_ is the quantum conductance, *g* is the Landé factor, and μ_B_ is the Bohr magneton constant. We find *G*_↑↓_ = 5.56 × 10^13^ Ω^–1^ m^–2^, comparable to reported Pt/YIG spin mixing conductance values.^34−36^

Figure 1d shows a schematic of the circuit used to characterize the auto-oscillations in a Pt nanowire (200 nm width by 400 nm length) patterned on an LAFO thin film. The auto-oscillation signal is measured above the noise floor of −128 dBm with a 1 MHz bandwidth. Figure 1e shows line scans of the auto-oscillation signal at a fixed *I*_DC_ = 1.4 mA for several applied fields *H*_ext_. The external field is applied at ϕ = 70°, an intermediate angle between 45°, where the resistance change due to spin-Hall magnetoresistance is maximized, and 90°, where the spin torque efficiency is highest. As expected, the auto-oscillation frequency increases with *H*_ext_. From Figures 1d and 2b,d, we can clearly observe two critical onset fields: (1) The first onset field appears around 28 mT when the auto-oscillation signal is first detectable with the spectrum analyzer. (2) The second onset field appears around 40–45 mT when the auto-oscillation frequency begins to depend linearly on the applied field. This auto-oscillation mode transition is associated with the 47.1 mT in-plane anisotropy field of the LAFO thin film, as shown in Figure 1b. Below this anisotropy field, the LAFO film is unsaturated and its dispersion curve is nonlinear. Once the external field is stronger than the in-plane anisotropy field, the LAFO film is saturated and the dispersion curve becomes a linear function of the field. The peak is broad at the onset field and then narrows to a single peak when the oscillation frequency increases linearly with the field. By fitting to a Lorentzian function, we determine that the linewidth of the PSD signals decreases from 84 to 17 MHz when *H*_ext_ increases from 40 to 80 mT and find a maximum quality factor of 523.

**Figure 2:**
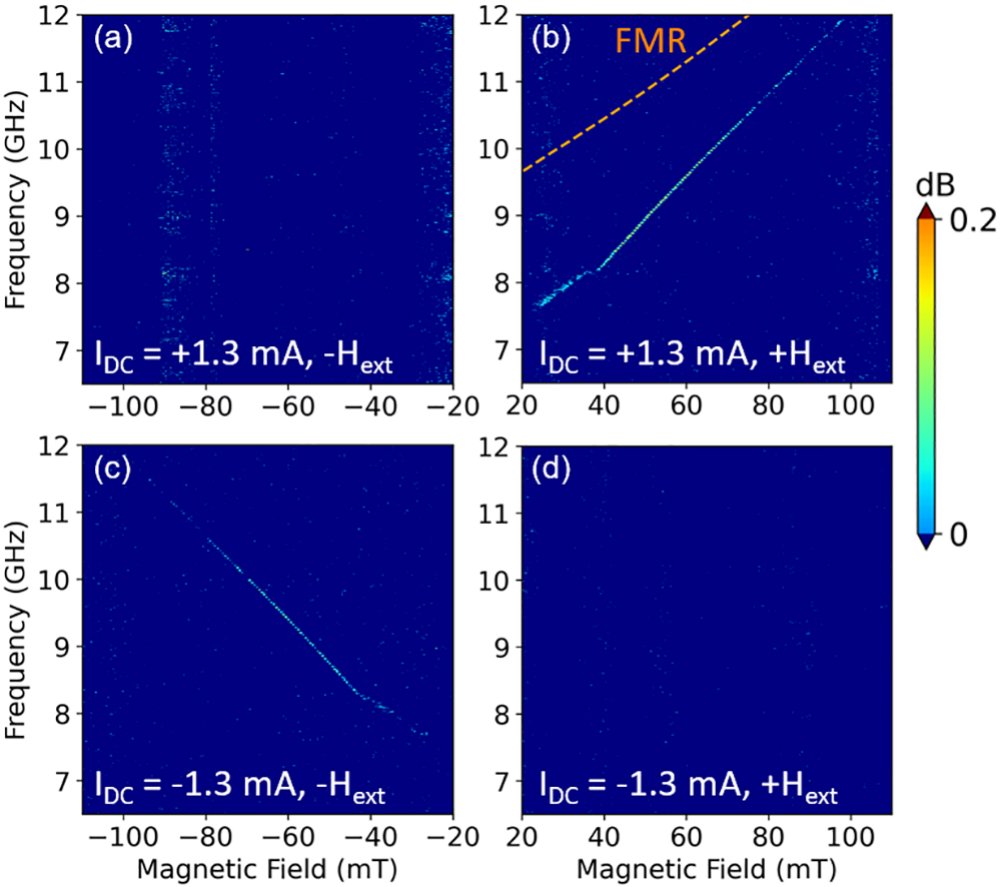
PSD color maps of a Pt/LAFO nanowire sample as a function of the frequency and field applied at ϕ = 70° for the four different current field polarity combinations. When *I*_DC_ and *H*_ext_ have the same polarity (panels b and c), an auto-oscillation signal is detected. However, no auto-oscillation signal is seen in panels a and d when they have opposite polarity. The orange line in panel b shows the FMR frequency-resonance field dependence of Pt/LAFO thin films.

Power spectrum density (PSD) map scans are conducted to investigate this auto-oscillation signal’s origin. Figure 2 shows the PSD map of the auto-oscillation amplitude as a function of the frequency and *H*_ext_ in four quadrants, where different polarities of the electric current *I*_DC_ and *H*_ext_ directions are represented. A direct current (DC) of 1.3 mA with different polarities is applied. When *I*_DC_ and *H*_ext_ have the same polarity, the SHE-induced spin current in Pt can inject spin angular moment at the Pt/LAFO interface that compensates for the damping, driving magnetic moments in the LAFO layer into auto-oscillations. The asymmetric signal in the four quadrants confirms that magnetization dynamics is mainly driven by the SHE-induced spin current instead of a temperature-gradient-induced spin Seebeck effect (SSE),^25,37^ which would be independent of the polarity of *I*_DC_ because the Seebeck effect is associated with Joule heating that goes at *I*_DC_^2^. Taking Figure 2b as an example, we observe that auto-oscillations begin around 20 mT with a broad peak and become linearly dependent on the applied field for fields exceeding 40 mT. A similar phenomenon occurs at the opposite polarity of the current and field. On top of the auto-oscillation dispersion curve in Figure 2b, the orange dash line represents the broadband FMR measurement of the same LAFO film. Clearly, the auto-oscillation dispersion curve is significantly red-shifted compared to the intrinsic dispersion curve of LAFO. To investigate the origin of the spectrum signal, we estimate the signal amplitude that originates from the inductive voltage and SMR-induced voltage (see Supplementary Note 3). The upper bound ratio of the two signals *V*_*x*_^Ind^/*V*_*x*_^SMR^ ≃ 0.12, showing that the spectrum signal is dominated by the SMR-induced voltage.

The current-swept PSD shows a similar behavior. Figure 3 shows the PSD map of auto-oscillation as a function of the bias current at a constant field of *H*_ext_ = 46.3 mT. After the onset current of 1.35 mA, the auto-oscillation frequency redshifts with increasing bias current. Because of the strong in-plane anisotropy field of LAFO, the Oersted field generated by the current is much smaller than its intrinsic anisotropy field, making it an unlikely root cause of the redshift. Instead, a redshift of the dispersion curve is a common characteristic of the excitation of localized spin-wave modes.^16,38^ In our study, this localized mode is achieved by concentrating the spin current in the small region of the Pt nanowire.^39^ Localized spin-wave excitations under the Pt nanowire are also seen in the micromagnetic simulations discussed in the next section.

**Figure 3:**
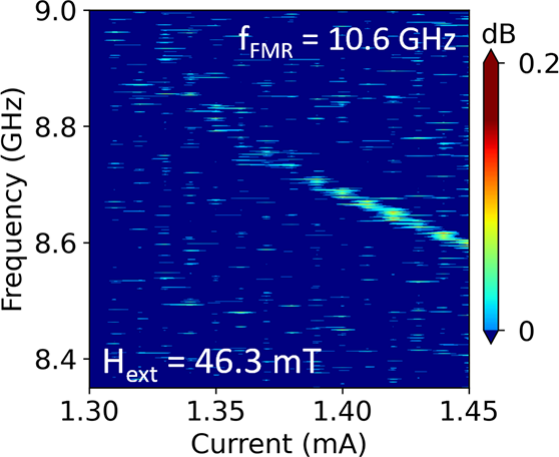
PSD map of auto-oscillations as a function of the current at a fixed *H*_ext_ = 46.3 mT and an angle of ϕ = 70°. The FMR frequency is 10.6 GHz for *H*_ext_ = 46.3 mT.

The transitions of the PSD signal from broad to narrow, as shown in Figure 2b,c, are related to the in-plane anisotropy of the LAFO thin film. At small *H*_ext_, when *H*_ext_ is comparable to *H*_a_, the linewidth of the microwave emission signal is broadened due to competition between the external field and the in-plane anisotropy field. This suggests that multiple auto-oscillation states may be excited simultaneously in the low-field region.^40,41^

The current-swept PSD map in Figure 3 shows a similar behavior. The onset *I*_DC_ to drive auto-oscillations in Pt/LAFO is 1.39 mA, corresponding to a current density of 1.1 × 10^12^ A m^–2^, which is comparable to the threshold current for driving auto-oscillations in a Py/Pt nanowire oscillator.^16^ At the onset *I*_DC_, the signal linewidth is broad, which is likely due to the phase noise and mode hopping that limits signal coherence.^40−42^ Above the onset current, the linewidth of the auto-oscillation peak becomes narrower as *I*_DC_ increases, suggesting a more coherent oscillation state. Unlike other studies^23,25,26^ where auto-oscillations occur only at low frequency, in the Pt/LAFO heterostructures, auto-oscillation signals occur at high frequencies, from 7 GHz to more than 12 GHz, without significant signal degradation. LAFO possesses a strong in-plane anisotropy that significantly enhances *M*_eff_ and thus increases the FMR frequency. We estimate the spin-precession cone angle from the measured microwave power emission in Figure 2. The integrated power *P*_int_ is proportional to *I*_DC_^2^ and the amplitude of the resistance oscillations.^8,13^ At *H*_ext_ = 80 mT, the integrated power generated by the SHNOs is 0.21 fW, giving a spin-precession angle of approximately 2.4°. The maximum microwave power output per unit DC input of Pt/LAFO is comparable to other types of spin–orbit torque oscillators (see Supplementary Note 4).^16,23^

To understand the nature of the excited spin-wave modes in the Pt/LAFO heterostructures, we used MuMax^3^^43^ to perform micromagnetic simulations and compare the results with the measured FMR, ST-FMR, and auto-oscillation power spectrum. The simulated SHNO microwave emission is presented in Figure 4a in a color map as a function of the frequency and *H*_ext_. The bilayer system is subjected to *I*_DC_ = 1.3 mA and *H*_ext_ at ϕ = 70° at *T* = 0 K. On top of the simulated PSD spectrum in Figure 4a are the experimental results from FMR, ST-FMR, and spectrum analyzer measurements. The simulation result shows two auto-oscillation modes: one at higher frequency and one at lower frequency. The measured auto-oscillation signals (green points in Figure 4a) agree well with the simulation result in the high-frequency region and have a slight deviation in the low-frequency region. The broad linewidth at low frequency suggests spin-wave channel competition and mode hopping.^40,41^Figure 4b shows the mode profile of the LAFO layer at *H*_ext_ = 80 mT obtained by taking the cell-wise fast Fourier transform of time evolution of the magnetization. The excited spin-wave mode is localized beneath the active spin injection region and extends in the directions perpendicular to the direction of *H*_ext_. In Figure 4a, the fact that the auto-oscillation frequencies are much less than the FMR frequencies also confirms that the excited spin-wave modes are localized.

**Figure 4:**
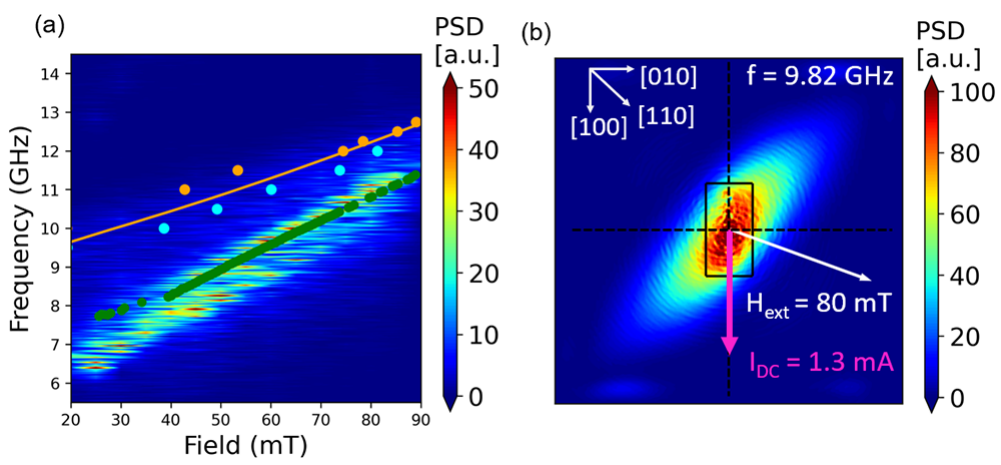
(a) Micromagnetic simulation of an auto-oscillation PSD color map. Experimental data points are shown on the color map for comparison. The orange points are the FMR data with *H*_ext_ applied along the [100] direction. The orange line is a fit to the Kittel model. The cyan points are the ST-FMR results at zero bias current. The green points are the auto-oscillation signal of a nanowire sample obtained from the spectrum analyzer. (b) Spatial profile of the mode at 9.82 GHz and *H*_ext_ = 80 mT along ϕ = 70° showing that the mode is mainly localized in the spin-injection region. The dashed lines indicate the hard axes of LAFO.

We now comment on possible reasons for the auto-oscillation signals being successfully detected in Pt/LAFO. From the Pt/LAFO SMR, we determined that Pt/YIG and Pt/LAFO interfaces have comparable spin-mixing conductance. The auto-oscillation precession angle in Pt/LAFO was found to be approximately 2.4°, which is on the same order as that reported in SHE and spin-Seebeck-generated auto-oscillations in Pt/YIG: 1–3°^23,44^ and 6°.^25^ A key factor contributing to this distinction may be the unique magnetic properties of LAFO, which result in significant nonlinear frequency shifts of the auto-oscillation mode relative to the FMR mode. Compared to YIG, LAFO exhibits stronger in-plane and cubic magnetic anisotropies at the origin of the larger nonlinearity.

In summary, our work presents the electrical detection of coherent auto-oscillation signals in an SHNO consisting of a Pt nanowire on top of an unpatterned LAFO thin film. We observed emitted microwave power originating from ISHE, with narrow linewidths. The current and magnetic-field-dependent PSD map proves that the auto-oscillation state is excited by the SHE, and the redshift of the dispersion curves indicates that the auto-oscillation is a localized spin-wave mode. Micromagnetic simulations suggest that the broad linewidths at low *H*_ext_ are likely due to mode hopping and show a localized mode at high *H*_ext_. We believe that it is the complex magnetic energy landscape, including the large in-plane magnetic anisotropy and cubic anisotropy of LAFO, that makes it an ideal candidate to observe nonlinear processes. Our work shows that a Pt nanowire/LAFO thin-film heterostructure is a promising platform for SHNO devices and opens new possibilities for coupled SHNOs for neuromorphic computing and magnonics applications.

## Supporting Information Available

Additional details on the experimental methods and micromagnetic simulations as well as supporting data and theoretical analysis, including an estimation of the auto-oscillation precession angle based on spectrum analyzer measurements, further analysis on the relative contributions of the SMR and inductive voltage signals, demonstrating that the observed signal is primarily due to SMR, and a comparative summary of the spin-torque oscillator properties, providing context for the performance of the Pt/LAFO heterostructure in relation to other spin-Hall nanooscillators (PDF)
