# Supplementary material for: Electrical Detection of Spin-Hall-Induced Auto-oscillations in Lithium Aluminate Ferrite Thin Films
Source: Nano Lett. 2025 Apr 10;25(16):6399–404. doi: 10.1021/acs.nanolett.4c06305 (PMC12023014; doi:10.1021/acs.nanolett.4c06305)
Supplement: Supplementary file 1 — nl4c06305_si_001.pdf [file nl4c06305_si_001.pdf]

Supplementary Materials: Electrical detection of spin Hall induced auto-oscillations in lithium aluminate ferrite thin films

Haowen Ren,[1,*] Ya-An Lai,[1,†] Sanyum Channa,[2,3] Daisy A. O'Mahoney,[3,4] Xin Yu Zheng,[3,5] Yuri Suzuki,[3,5,‡] and Andrew D. Kent[1,§]

[1] *Center for Quantum Phenomena, Department of Physics, New York University, New York, NY 10003, USA*

[2] *Department of Physics, Stanford University, Stanford, CA 94305, USA*

[3] *Geballe Laboratory for Advanced Materials, Stanford University, Stanford, CA 94305, USA*

[4] *Department of Materials Science and Engineering, Stanford University, Stanford, CA 94305, USA*

[5] *Department of Applied Physics, Stanford University, Stanford, CA 94305, USA*

Supplementary Note 1. Methods

A. Sample Preparation and Measurements

Epitaxial $Li_{0.5}Al_{0.5}Fe_2O_4$ (LAFO) films were deposited on (001) $MgAl_2O_4$ (MAO) substrates via pulsed laser deposition, following the procedure previously reported [1]. After we obtained the LAFO ferrite thin film, we patterned 200 nm wide nanowires by first spin-coating with PMMA 495 4A and then exposing by an Elionix 50keV E-beam lithography system. A 6 nm Pt thin film was then deposited via magnetron sputtering on top of the LAFO layer and immediately lifted-off after the deposition to form the shape of nanowire. Cr/Au contact pads were then deposited and used to define the effective length of the Pt nanowire. The gap between two contact pads was fixed at 400 nm. A schematic drawing of the fabricated device is shown in Fig. 1(d). SMR measurements were conducted on micron-scale wires ($2 \times 5.8\ \mu m^2$) mounted on a PPMS rotator. The measured resistance of the sample is about 258 Ω. During the measurement, a current of 500 μA is used and a constant magnetic field of 200 mT is applied to ensure that the sample magnetization and field are aligned. The sample is then rotated with respect to the external field. Ferromagnetic resonance (FMR) measurements were performed using a vector network analyzer (VNA) technique. Spin-torque ferromagnetic resonance (ST-FMR) measurements were performed using a field-modulated lock-in setup. During the ST-FMR measurement, the field is applied at an angle $\phi = 70^\circ$ to the current, the same as the field angle applied in the PSD measurements. A high-resolution spectrum analyzer (Keysight N9030B) with an +29 dB low-noise amplifier was used for power spectrum density (PSD) mapping measurements. The resistance of the nanowire device is 403 Ω. The DC was injected into the nanowire using a bias-T. The RF signal was sampled with a spectrum analyzer connected to the high-frequency port of the bias-T. The magnetic properties before and after Pt deposition were characterized at room temperature by in-plane FMR measurements with an external field applied along the hard axis. The measured FMR spectra was fitted to a Lorentzian to extract the resonant fields and linewidths, which were then fitted to the Kittel equation.

* Co-author; haowren@gmail.com

† Co-author; audre.lai@nyu.edu

‡ ysuzuki1@stanford.edu

§ andy.kent@nyu.edu

B. Micromagnetic Simulations

The simulation setup is as follows. A $300 \times 300 \times 5$ mesh with a $5 \times 5 \times 5$ nm^3 cell size was used. An exponential damping with a maximum at the lateral boundaries was used to avoid spin wave reflections at the boundaries. Spin current was applied in the $400\times200\times5$ nm^3 Pt nanowire region. The following material parameters are used: Exchange stiffness $A = 4$ pJ/m, saturation magnetization $M_s = 200$ kA/m, 1st order cubic anisotropy constant $K_c = -5.5$ kJ/m^3, 1st order uniaxial anisotropy constant $K_u = -156$ kJ/m^3 and spin polarization efficiency $P = 0.15$. The direction of the cubic anisotropy points along the hard axis of LAFO and the uniaxial anisotropy is set to mimic the LAFO effective magnetization $\mu_0 M_{\text{eff}} = 1.8156$ T. With COMSOL modeling, we calculated the Oersted field spatial distribution due to $I_{\text{DC}} = 1.3$ mA and import the field to the MuMax^3 model. To account for the spin pumping effect, we set the Gilbert damping $\alpha = 0.01$ in the spin current application region and $\alpha = 0.004$ in the rest of the LAFO layer. The total simulation time is 150 ns. Fast Fourier transform (FFT) is performed on the spatially averaged magnetization time series with a sampling frequency of 100 MHz to extract the spin-wave frequencies. The spatial mode profile is obtained by performing cell-wise FFT.

Supplementary Note 2. Auto-oscillation Precession Angle

The auto-oscillation precession angle θ presented in the main text is estimated from the measured peak power at the spectrum analyzer, as the precession angle is related to the applied DC I_{DC}, the amplitude of the auto-oscillation resistance δR_{ac}, the device resistance R, and the circuit load R_{50} by the following equation [2–4]:

$$P = \frac{1}{2R_{50}}\big(I_{\text{DC}}\delta R_{\text{ac}}\frac{R_{50}}{R + R_{50}}\big)^2. \tag{1}$$

From the spectrum analyzer measurement at an external field $H_{\text{ext}} = 80$ mT and $I_{\text{DC}} =$ 1.3 mA as shown in Fig. 2(b), the peak power $P = 0.105$ dB is above the noise floor of -128 dBm. The maximum integrated power is $P = 0.21$ fW, which gives $\delta R_{\text{ac}} = 4.56 \times 10^{-4}\,\Omega$. As a result of the spin Hall magnetoresistance (SMR), the resistance of Pt/LAFO when the magnetization forms an angle ϕ with the current direction is:

$$R = R_0 + \frac{\Delta R}{2}\cos(2\phi). \tag{2}$$

From the SMR measurements shown in Fig. 1(a): $R_0 = 403\ \Omega$ and $\Delta R = -0.032\ \Omega$. During auto-oscillation, the oscillating resistance δR_{ac} is proportional to the precession angle θ and ΔR assuming uniform magnetization precession under the Pt nanowire:

$$\delta R_{\mathrm{ac}} = \theta \Delta R \sin(2\phi). \tag{3}$$

This gives an estimate of the precession angle of $\theta \approx 2.4°$.

Supplementary Note 3: Estimation of the Inductive Voltage

When applying a DC to induce auto-oscillations in the Pt/LAFO, the resulting signal measured by a spectrum analyzer can arise from multiple sources such as the spin Seebeck effect (SSE), spin Hall magnetoresistance (SMR), and an inductive signal. We have excluded SSE to be the main contribution due to the polarity-dependent auto-oscillation signal.

Here, we estimate the ratio of the inductive signal to that associated with SMR starting with the induced EMF associated with magnetization precession.

C. Inductive Signal

Faraday's law can be used to compute the inductive signal, which can be expressed as:

$$\mathcal{E} = -\frac{d\Phi}{dt},$$

where $\mathcal{E}$ is the induced EMF and $\Phi = \int_S \mathbf{B} \cdot d\mathbf{A}$ is the magnetic flux. To estimate the flux linking the electrical circuit, we consider the Pt/LAFO plane, which has a normal along the z-direction. An upper bound for this flux can be determined from the magnetic flux in the nanowire region that links this plane. That is, we determine the approximate fraction of magnetic flux in the area $A = \ell t$, where ℓ is the length of the nanowire and t that links the Pt/LAFO plane.

As the LAFO film is extended most of the magnetic field is contained in the LAFO, as the component of magnetization in the field direction is approximately constant. The

modulation of AC field is associated with the ellipicity of the magnetization orbits, given approximately by: $\eta = H/M_{\text{eff}} \sim 0.05$ at 10 GHz, $\mu_0 H = 80$ mT. Thus

$$\Phi(t) \simeq \frac{1}{2}\eta\mu_0 M_s\theta\sin(\omega t)\ell t. \tag{4}$$

And, the induced EMF is thus approximately:

$$\mathcal{E} \simeq \frac{1}{2}\eta\omega\mu_0 M_s\theta\cos(\omega t)\ell t. \tag{5}$$

D. SMR Signal

The SMR signal is given from Eq. 3 by:

$$V_{\text{SMR}} = I_{\text{DC}}\delta R_{\text{AC}}, = I_{\text{DC}}\Delta R\theta\sin(2\phi)\sin(\omega t).$$

E. Inductive to SMR signal ratio

Therefore, the ratio of the magnitude of induced EMF to SMR voltage is given by:

$$\frac{\mathcal{E}}{V_{\text{SMR}}} = \frac{\omega\eta\mu_0 M_s\theta\ell t}{2I_{\text{DC}}\Delta R\theta\sin(2\phi)}. \tag{6}$$

Or,

$$\frac{\mathcal{E}}{V_{\text{SMR}}} = \frac{\pi f\eta\mu_0 M_s\ell t}{I_{\text{DC}}\Delta R\sin(2\phi)}. \tag{7}$$

Taking $\mu_0 M_s = 0.25$ T, $f = 10$ GHz, $\ell = 400$ nm, $t = 20$ nm, $\phi = 70°$, $I_{\text{DC}} = 1.3$ mA and $\Delta R = 32$ mΩ gives

$$\frac{V_x^{\text{Ind}}}{V_x^{\text{SMR}}} \simeq 0.12. \tag{8}$$

We thus conclude that the inductive signal is at least a factor of eight times smaller than the SMR signal.

Supplementary Note 4: Property of Spin-Torque Oscillators

Summary of properties of spin-torque oscillators including a Pt nanowire on an extented LAFO film (Pt/LAFO, this work), Pt/YIG microdiscs, and Pt/Py nanowires.

Sample	α	M_{eff} (MA/m)	Onset Frequency (GHz)	Threshold Current Density ($\times 10^{12}$ A/m^2)	Precession Angle (°)	Power/DC Current (pW/A)
Pt/LAFO (This work)	0.0042	1.5	7.6	1.1	2.36	0.16
Pt/YIG Microdiscs [4, 5]	0.0020, 0.0013	0.17	1	0.11-0.3	1, 3.5	0.18
Pt/Py NW [6]	0.026	0.62	5	1.2	0.06	0.13

Supplementary Table S1. Summary of the spin-torque oscillator properties.

References

[1] X. Y. Zheng, S. Channa, L. J. Riddiford, J. J. Wisser, K. Mahalingam, C. T. Bowers, M. E. McConney, A. T. N'Diaye, A. Vailionis, E. Cogulu, H. Ren, Z. Galazka, A. D. Kent, and Y. Suzuki, Ultra-thin lithium aluminate spinel ferrite films with perpendicular magnetic anisotropy and low damping, Nature Communications **14**, 4918 (2023).

[2] C. Safranski, I. Barsukov, H. K. Lee, T. Schneider, A. Jara, A. Smith, H. Chang, K. Lenz, J. Lindner, Y. Tserkovnyak, M. Wu, and I. Krivorotov, Spin caloritronic nano-oscillator, Nature Communications **8**, 117 (2017).

[3] S. I. Kiselev, J. Sankey, I. Krivorotov, N. Emley, R. Schoelkopf, R. Buhrman, and D. Ralph, Microwave oscillations of a nanomagnet driven by a spin-polarized current, Nature **425**, 380 (2003).

[4] Z. Duan, A. Smith, L. Yang, B. Youngblood, J. Lindner, V. E. Demidov, S. O. Demokritov, and I. N. Krivorotov, Nanowire spin torque oscillator driven by spin orbit torques, Nature Communications **5**, 5616 (2014).

[5] M. Collet, X. De Milly, O. d'Allivy Kelly, V. V. Naletov, R. Bernard, P. Bortolotti, J. Ben Youssef, V. Demidov, S. Demokritov, J. L. Prieto, M. Muñoz, V. Cros, A. Anane, G. de Loubens, and O. Klein, Generation of coherent spin-wave modes in yttrium iron garnet microdiscs by spin–orbit torque, Nature Communications **7**, 10377 (2016).

[6] H. Ren, X. Y. Zheng, S. Channa, G. Wu, D. A. O'Mahoney, Y. Suzuki, and A. D. Kent, Hybrid spin Hall nano-oscillators based on ferromagnetic metal/ferrimagnetic insulator heterostructures, Nature Communications **14**, 1406 (2023).
